# Bioassay-Guided Isolation of Broad-Spectrum Fungicidal Active Compound from *Artemisia ordosica*

**DOI:** 10.3390/metabo11090629

**Published:** 2021-09-17

**Authors:** Gaijuan Tang, Shuyu Yang, Wenqiong Hu, Jingyi Jiang, He Yan, Juntao Feng, Chao Zhang, Yonghong Wang

**Affiliations:** 1 College of Plant Protection, Northwest A&F University, Yangling 712100, China; tangrui-happy@163.com (G.T.); shuyu3287@163.com (S.Y.); huwenqion_g@163.com (W.H.); jiangjingyi@nwafu.edu.cn (J.J.); yh-run@163.com (H.Y.); fengjt67@hotmail.com (J.F.); 2 Shaanxi Research Center of Biopesticide Engineer & Technology, Northwest A&F University, Yangling 712100, China; 3 College of Agronomy, Northwest A&F University, Yangling 712100, China

**Keywords:** *Artemisia ordosica*, natural products, trans-dehydromatricaria ester, broad-spectrum, botanical fungicides

## Abstract

To avoid the widespread resistance of commercial fungicides, new broad-spectrum botanical fungicides need to be developed. In previous bioactive screening assays, extracts of *Artemisia ordosica* Krasch. (*A. ordosica*) had highly antifungal activities, but the responsible phytochemicals were unidentified. In this study, active compounds of *A. ordosica* extracts were identified using a bioassay-guided method, and antifungal assays were performed in vitro and in vivo. The bioactive compounds were dissolved in petroleum ether, and the best antifungal fraction contained four compounds: trans-dehydromatricaria ester (TDDE), 7, 4-demetylnringenin, capillarin, and stearic acid. Among them, TDDE exhibited the highest antifungal activity against six pathogenic fungi and five bacteria. It exhibited significant fungicidal activity against *Thanatephorus cucumeris* and *Botrytis cinerea* with EC_50_ values of 0.464 μg/mL and 1.4 μg/mL, respectively. The living tissue bioassay results showed that the relative protection effects (RPE) of TDDE on tomato leaves, tomato fruit, and strawberry leaves infected with *B. cinerea* reached 76.78%, 86.2%, and 80.89%, respectively. In pot experiments, the RPE on tomato and strawberry plants infected with *B. cinerea* reached 84.11% and 96.37%, respectively. Morphological and physiological examination showed that TDDE had significant inhibitory effects on mycelial growth, including increased top offshoot, contorted hyphal tips, and extravasated cytochylema. Meanwhile, bactericidal activities of TDDE were significantly higher than kanamycin and streptomycin in five bacteria, and the plant tissue experiments further demonstrated that it had an 88.31% RPE on walnut leaves infected with *Xanthomonas campestris* pv. *jugiandis*, 72.18% RPE on potato infected with *Erwinia carotovora* subsp. *carotovora*, and 82.50% RPE on kiwifruit branches infected with *Pseudomonas syringae* pv. *actinidiae*. The active compounds isolated from *A. ordosica* in this study show great potential value for developing broad-spectrum fungicides, and also provide an important way to identify and isolate new bioactive products from medicinal plants.

## 1. Introduction

Global industrial crops are threatened by numerous pathogenic fungi and bacteria, and incalculable economic losses result every year [1,2,3]. Currently, chemical control is still the most efficient method to control pathogenic microorganisms; however, extensive drug resistance has developed in pathogenic microbe populations due to unscientific and long-term pesticide usage [4,5,6]. Therefore, there is great practical significance and urgent demand for developing new broad-spectrum fungicides [7]. Traditional Chinese herbal medicinal crops are considered a giant treasure trove in the search for environmentally friendly botanical fungicides with low cost, high efficacy, and easy degradation [8,9].

*Artemisia ordosica* Krasch is a representative desert psammophyte and cultivated medicinal crop grown in the northwest of China [10]. The dried whole herb of *A. ordosica* was used as a traditional Chinese medicine (Mongolian medicine) for the treatment of rheumatic arthritis, colds, headaches, and sore throats [11,12]. It also has antioxidant activity [13] and high immunological activity [14], and reduces bilirubin alcohol, hypoglycemia, antistain selection, and anti-inflammation [12,15]. The chemical constituents of *A. ordosica* are abundant and diverse, including flavonoids [16], terpenes [17,18], polysaccharides, sterols [19], coumarins [20], phenylpropanoids [17], organic acids, amino acids, and microelements [15]. In recent years, some compounds have been isolated, including a new lignan glycoside (artemordolignan glycoside A) [21], a new coumarin having a new skeleton (arteordocoumarin A) [22], four new compounds (arteordoyn A, arteordoyn B, arteordosin A, and arteordosin B) [23,24], and five polyacetylenes (capillene, capillinol, capillin, trans-dehydromatricaria ester, and cis-dehydromatricariaria ester) [25].

After isolation, some compounds and crude essential oils exhibit reasonable repellent and fumigant activity [25]. For instance, arteordosin A and B showed antioxidant activities and could be potentially used as an antihyperlipidemic agent in routine clinical practice [23]. The inhibition rates of *A. ordosica* acetone extract against Fusarium oxysporum, *Colletotrichum lagenarium*, *Botrytis cinerea*, and *Alternaria solani* were more than 70%. Among them, *B. cinerea* is a major phytopathogen that affects a broad range of plant species. As a necrotrophic pathogen with a broad host range, *B. cinerea* is difficult to control [26]. The inhibition rate of *A. ordosica* acetone extract against *B. cinerea* reached 100%, and the protective effect was 71.23% [27]. However, the specific bioactive compounds responsible for this fungicidal activity are unknown.

*A. ordosica* has abundant metabolites with various biological activities, providing a broad range of prospects to be developed as botanical fungicides. In screening bioactive materials from Chinese medicinal herbs for new agrochemicals, significant antifungal activities of the crude extract from the dried and ground aerial parts of *A. ordosica* were found, but the bioactive compounds are still unidentified. In the present study, we followed a bioassay-guided method to isolate active ingredients from *A. ordosica* extracts and investigated their antifungal and antibacterial activities in vitro and in vivo, establishing an effective foundation for developing new botanical fungicides.

## 2. Results

### 2.1. Bioassay-Guided Isolation

**Figure 1:**
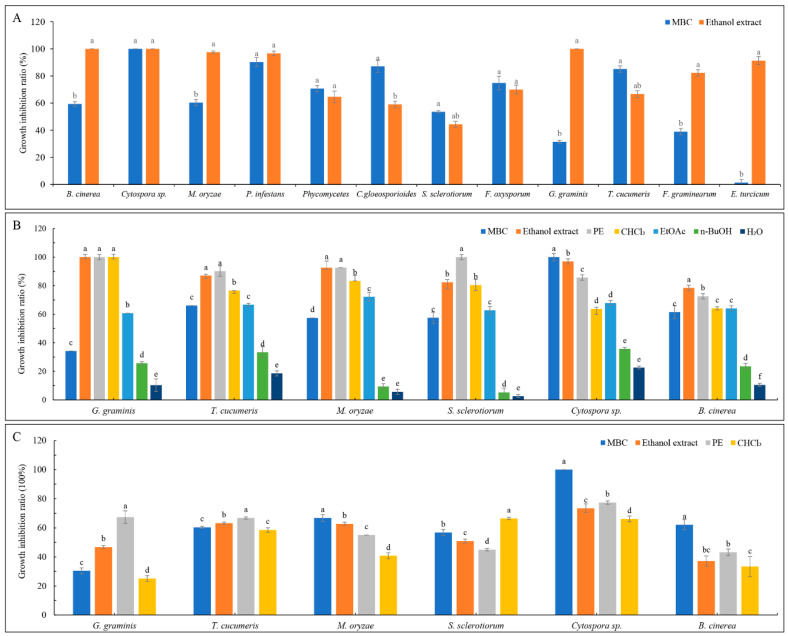
Antifungal activities of different extracts isolated from *A. ordosica*. (**A**) The antifungal activities of ethanol crude extracts at a concentration of 1000 μg/mL; (**B**) the antifungal activities of Petroleum Ether (PE), Chloroform (CHCl_3_), Ethyl Acetate (EtOAc), and n-BuOH extraction sections isolated from ethanol crude extracts at a concentration of 500 μg/mL; and (**C**) the antifungal activities of PE, CHCl_3_, EtOAc, and n-BuOH extracts at a concentration of 200 μg/mL. MBC, methyl 2-benzimidazole carbamate (0.5 μg/mL). Different lowercase letters in the figures indicate significant differences at *p* < 0.05 among different extracts against the same strain.

The dried and ground aerial parts of *A. ordosica* were extracted with 80% ethanol at room temperature till exhaustion. The antifungal activities of ethanol extracts were determined in vitro with 12 plant pathogens and exhibited inhibition rates greater than 60% for all except *S. sclerotiorum* and *C. gloeosporiodes*; the extracts completely inhibited the mycelia growth of *Cytospora* sp., *B. cinerea*, and *G. gramin* is, and also displayed high fungicidal activities to *M. oryzae*, *P. infestans*, *G. graminis*, *T. cucumeris*, *F. oxysporum*, and *E. turcicum* (Figure 1A). Compared to MBC (methyl 2-benzimidazole carbamate; carbendazim), the ethanol extracts had significant antifungal activities to *B. cinerea*, *M. oryzae*, *G. graminis*, *F. oxysporum*, and *E. turcicum* (Figure 1A), demonstrating the great potential of *A. ordosica* ethanol extracts in botanical pesticide exploitation.

The antifungal activities of the PE, CHCl_3_, EtOAc, and n-BuOH extracts isolated from ethanol extracts were evaluated. Under the concentration of 500 μg/mL, the PE extract had similar or higher fungicidal activities than the ethanol crude extracts against *G. graminis*, *T. cucumeris*, *M. oryzae*, and *S. sclerotiorum* (Figure 1B). To further determine the extracts with the greatest activity, the concentration was reduced to 200 μg/mL, and the results showed that the PE extracts had better activity against *G. graminis*, *T. cucumeris*, and *Cytospora* sp. (Figure 1C). To obtain active compounds more efficiently and accurately, PE extract was used to determine the susceptibility of 10 different plant pathogens, and the results indicated that the antifungal activities on *B. dothidea*, *T. cucumeris*, and *G. graminis* were best, with the EC_50_ values of 75.007, 73.443, and 104.678 μg/mL, respectively (Table 1). *T. cucumeris* was selected as the bioassay-guided indicator in this experiment because of its faster growth characteristic.

**Figure 2:**
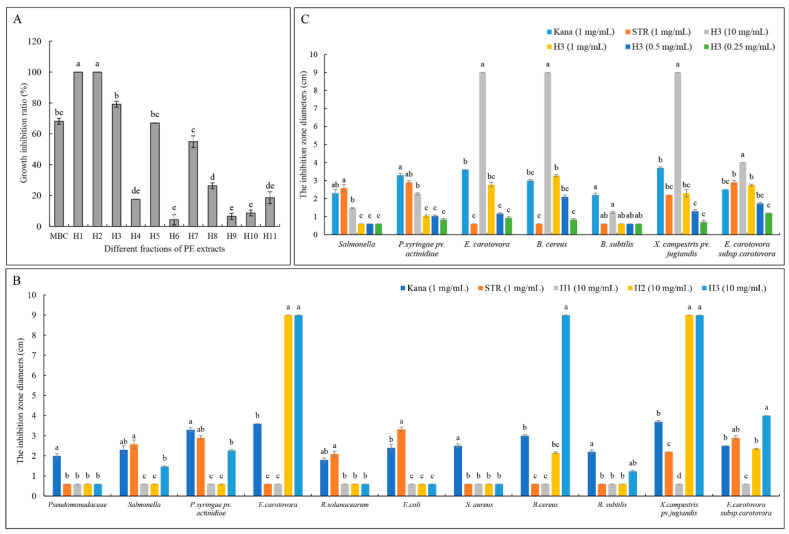
Antifungal and antibacterial activities of the different fractions of PE extracts. (**A**) Antifungal activities against *T. cucumeris* at a concentration of 100 μg/mL; (**B**) antibacterial activities of H1-3 fractions at a concentration of 10 mg/mL; and (**C**) antibacterial activities of H3 fractions at 0.25 to 10 mg/mL. MBC, methyl 2-benzimidazole carbamate (0.5 μg/mL); Kana, kanamycin (1.0 mg/mL); STR, streptomycin (1.0 mg/mL). Different lowercase letters in the figures indicate significant differences at *p* < 0.05 among different fractions against the same strain.

The PE extract was applied to a silica gel column (eluent: PE/Acetone) and 11 fractions were obtained (H1 to H11). H1 and H2 completely inhibited the growth of *T. cucumeris*, followed by H3 (Figure 2A). To screen broad-spectrum active compounds, the antibacterial activities of these three fractions against 11 different bacteria were performed, demonstrating that H3 (at 10, 1, 0.5, and 0.25 mg/mL) had the strongest inhibitory effects against several bacteria, including *P. syringae* pv. *actinidiae*, *E. carotovora*, *X. campestris* pv. *Jugiandis*, and *E. carotovora* sub sp. *carotovora*. (Figure 2B,C). Given the combined activities against fungi and bacteria, we inferred that the H3 fraction contained the broad-spectrum active compounds.

### 2.2. Structural Analysis of Bioactive Compounds

The active fraction H3 was isolated in this study, and four compounds were obtained and identified using Brucker DRX-500 MHz NMR.

**Figure 3:**
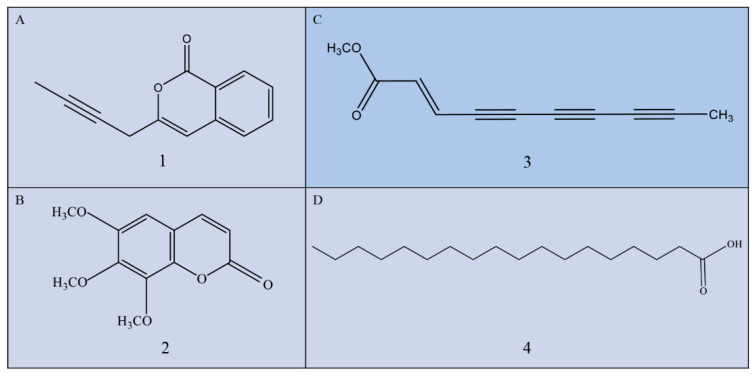
Chemical structure of isolated compounds from *A. ordosica*. (**A**) Capillarin (C_13_H_10_O_2_); (**B**) 6, 7, 8-trimethoxycoumarin (C_12_H_12_O_5_); (**C**) trans-dehydromatricaria ester (TDDE, C_11_H_8_O_2_); and (**D**) stearic acid (C_18_H_36_O_2_). The H-NMR and C-NMR of TDDE are provided in the Figure S1.

Compound **1** (Figure 3A, 50 mg) is a colorless crystal obtained after repeated recrystallization. Melting point is 123–125 °C, molecular formula is C_13_H_10_O_2_, and formula weight is 198. It is soluble in CHCl_3_, and the nuclear magnetic data are classified as follows: 1H-NMR (500 MHz, CDCl_3_) δ: 8.2 (1H, d, J = 8.0 Hz, 3-H), 7.7 (1H, dd, J = 15.2 Hz, 1.2 Hz, 5-H), 7.5 (1H, dd, J = 15.2 Hz, 1.2 Hz, 4-H), 7.4 (1H, d, J = 7.6 Hz, 6-H), 6.6 (1H, s, 8-H), 3.5 (2H, ms, 10-H), 1.1 (3H, s, 13-H), ^13^C-NMR (126 MHz, CDCl_3_) δ: 162.4 (C-1), 153.8 (C-9), 137.3 (C-7), 134.9 (C-5), 129.6 (C-3), 127.9 (C-4), 125.4 (C-6), 120.1 (C-2), 103.1 (C-8), 79.9 (C-12), 71.8 (C-11), 23.9 (C-10), 3.6 (C-13). Compound **1** was identified as capillarin according to its nuclear magnetic data [20].

Compound **2** (Figure 3B, 30 mg) is a white powder, molecular formula is C_12_H_12_O_5_, and formula weight is 236. It is soluble in CHCl_3_, and the nuclear magnetic data are classified as follows: 1H-NMR (500 MHz, CDCl_3_) δ:7.8 (1H, d, J = 9.5 Hz, H-4), 6.7 (1H, s, H-5), 6.3 (1H, d, J = 9.6 Hz, H-3), ^13^C-NMR (126 MHz, CDCl_3_), 4.1 (3H, s, -OCH_3_), 4.0 (3H, s, -OCH3), 3.9 (3H, s, -OCH3), δ:160.7, 157.5, 151.5, 149.3, 139.6, 138.2, 112.7, 107.0, 96.6, 62.4, 61.3, and 57.0. Compound **2** was identified as 6, 7, 8-trimethoxycoumarin according to its nuclear magnetic data [28].

Compound **3** (Figure 3C, 50 mg) is a pink powder, molecular formula is C_11_H_8_O_2_, and formula weight is 172. It is soluble in CHCl_3_, and the nuclear magnetic data are classified as follows: 1H-NMR (400 MHz, CDCl_3_) δ: ^1^H NMR (500 MHz, CDCl_3_) δ: 6.8 (d, J = 15.8 Hz, H-2), 6.4 (s, 1H, d, J = 15.8 Hz, H-3)), 3.8 (3H, s, H-1′), 2.1 (3H, s, H-10); ^13^C NMR (126 MHz, CDCl_3_) δ:165.7 (C-1), 133.6 (C-2), 123.6 (C-3), 83.1 (C-4), 80.6 (C-5), 71.6 (C-6), 71.1 (C-7), 64.7 (C-8), 58.2 (acetylenic carbons), 52.1 (C-1′), 4.8 (C-10). Compound **3** was identified as trans-dehydromatricaria ester (TDDE) according to its nuclear magnetic data (Figure S1) [25].

Compound **4** (Figure 3D, 35 mg) is a white pearlescent scale, molecular formula is C_18_H_36_O_2_, and formula weight is 284. It is soluble in CHCl_3_, acetone, and EtOH, and the nuclear magnetic data are classified as follows: 1H-NMR (400 MHz, CDCl_3_) δ: ^1^H NMR (500 MHz, CDCl_3_) δ: 2.4 (t, J = 7.1 Hz, 2H), 1.9-1.5 (m, 2H), 1.3 (s, 42H), 0.9 (t, J = 5.7 Hz, 4H); ^13^C NMR (126 MHz, CDCl_3_) δ: 180.0 (C-1), 34.1 (C-2), 32.0, 29.7, 29.6, 29.5, 29.4, 29.3, 29.1, 24.7, 22.7, 14.1, 1.0. Compound **4** was identified as stearic acid according to its nuclear magnetic data.

### 2.3. Antifungal Activity of TDDE (Compound **3**) In Vitro and In Vivo

#### 2.3.1. Effect of TDDE on the Mycelial Growth of Different Pathogens

**Figure 4:**
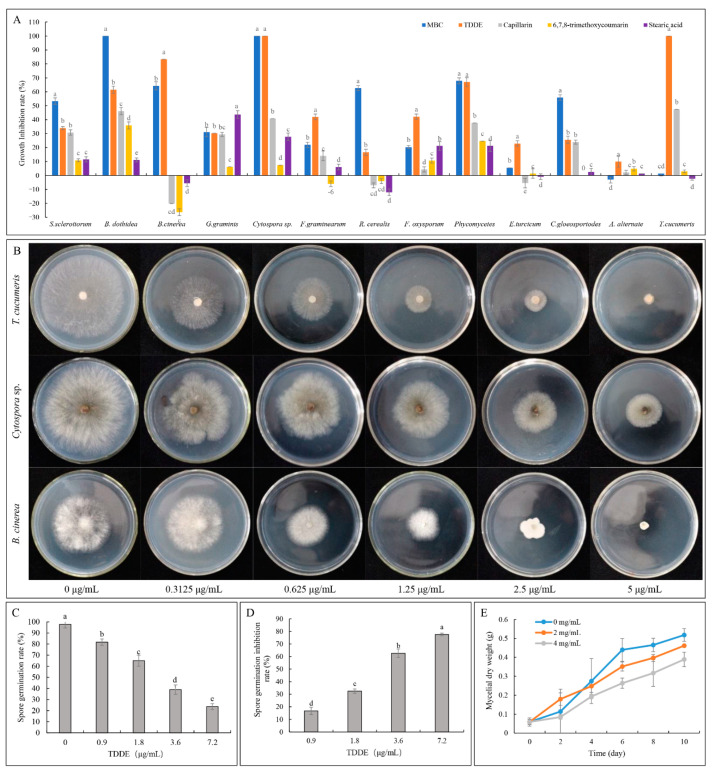
Antifungal activities of isolated compounds on different pathogenic fungi. (**A**) Antifungal activities of isolated compounds at a concentration of 50 μg/mL. MBC, methyl 2-benzimidazole carbamate (0.5 μg/mL); (**B**) the inhibitory effects of different concentrations of TDDE; (**C**) spore germination rates; (**D**) spore germination inhibition rate; and (**E**) mycelial dry weight. Different lowercase letters in Figure 4A indicate significant differences at *p* < 0.05 among different compounds against the same strain.

The antifungal activities against 13 pathogenic fungi were measured in culture, and the results showed that the activity of TDDE (Compound **3**) was significantly higher than the other three compounds tested at a concentration of 50 μg/mL (Figure 4A). The growth inhibition rates of *T. cucumeris* and *Cytospora* sp. were 100%, and the growth inhibition rates of *B. dothidea, B. cinerea, R. cerealis*, and *Phycomycetes* reached over 50% (Figure 4A). It should be noted that the other three compounds (6, 7, 8-trimethoxycoumarin, capillarin, and stearic acid) promoted the growth of *R. cerealis* and *B. cinerea* (Figure 4A). Based on the results of these experiments, toxicity curves of TDDE against *T.cucumeris*, *Cytospora* sp., *B. cinerea*, *B. dothidea*, *Phycomycetes*, and *G. graminis* were constructed, showing that TDDE displayed high fungicidal activities with EC_50_ values of 0.464 μg/mL in *T. cucumeris*, 1.722 μg/mL in *Cytospora* sp, and 1.401 μg/mL in *B. cinerea* (Figure 4B and Table 2).

#### 2.3.2. Effect of TDDE on *B. cinerea* Spore Germination

Spore germination of *B. cinerea* was studied under different concentrations of TDDE. The results showed that spore germination rates significantly decreased with increasing TDDE concentration, and the inhibition rate reached 77.53% at a concentration of 7.2 μg/mL (Figure 4C,D). The IC_50_ (half maximal inhibitory concentration) of TDDE against *B. cinerea* spores was 2.7 μg/mL (the virulence curve is Y = 3.313 − 1.429X, Chi-square value is 14.70, correlation coefficient R squared is 0.9818), which was higher than the EC_50_ against mycelia (1.401 μg/mL; Table 2), indicating that mycelial growth of *B. cinerea* was more sensitive to TDDE than spore germination. Furthermore, the mycelial dry weights in TDDE treatment groups were measured, and they were significantly lower than in the control group (Figure 4E), which also demonstrated that *B. cinerea* mycelial growth was significantly inhibited by TDDE.

#### 2.3.3. Effect of TDDE on Detached Plant Tissues Infected with *B. cinerea*

**Figure 5:**
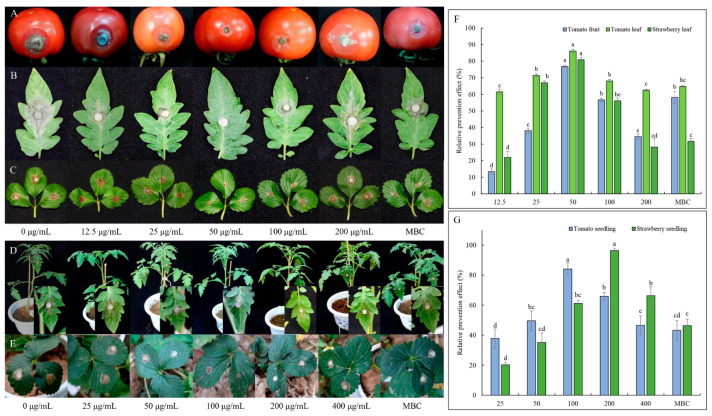
Relative prevention effects of TDDE against *B. cinerea*. (**A**–**E**) Tomato leaf, tomato fruit, strawberry leaf, tomato seedling, and strawberry seedling, respectively; (**F**) relative prevention effects of TDDE on plant tissues; and (**G**) relative prevention effects of TDDE on plants. MBC, methyl 2-benzimidazole carbamate (100 μg/mL). Different lowercase letters in the figures indicate significant differences at *p* < 0.05 among different concentration in the same tissue or seedling.

To further study the protective effect of TDDE on living tissue, the antifungal effect of this compound against *B. cinerea* was verified using a living tissue method in different plant tissues (tomato leaves, tomato fruit, and strawberry leaves). Under the concentration of 50 μg/mL, the protective effect of TDDE is optimal, and the relative prevention effect on tomato leaves, tomato fruit, and strawberry leaves reached 76.78%, 86.2% and 80.89%, respectively (Figure 5A–C,F).

#### 2.3.4. Effect of TDDE on Tomato and Strawberry Plants Infected with *B. cinerea*

In pot experiments, the optimal protection concentrations of TDDE on tomato and strawberry plants were 100 μg/mL and 200 μg/mL, respectively, and the relative prevention rates reached 84.11% and 96.37%, respectively (Figure 5D,E,G).

### 2.4. Effect of TDDE on the Mycelial Morphology of B. cinerea

**Figure 6:**
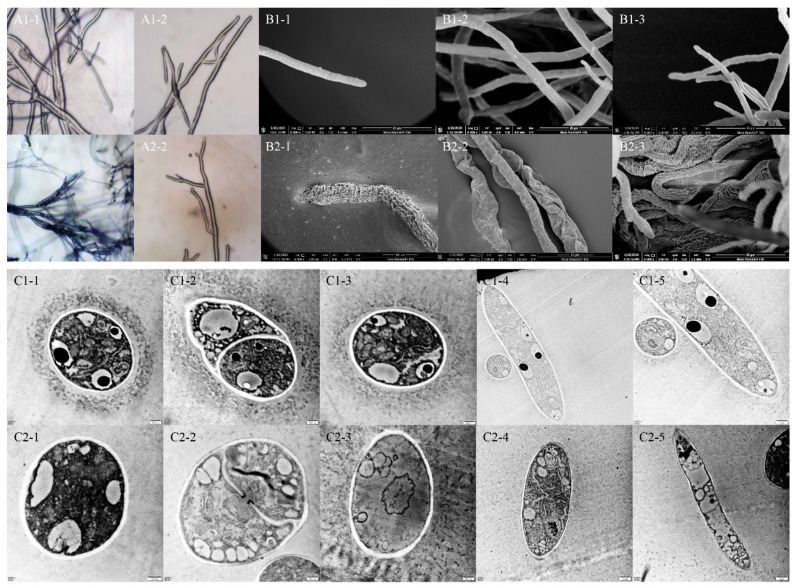
Effects of TDDE on microstructures of *B. cinerea*. Figure groups (**A**–**C**) show images taken with light microscope, scanning electron microscope (SEM), and transmission electron microscope (TEM), respectively. (**A1**–**C1**) represent blank controls, and (**A2**–**C2**) represent 1.4 µg/mL TDDE treatments. (**C1-1**–**1-3**) and (**C2-1**–**2-3**) show the cross sections of mycelia, the other images in group F show the vertical sections of mycelia.

The changes of mycelial morphology of *B. cinerea* were observed by light microscope, SEM, and TEM. According to the figures observed by light microscope, TDDE had an obvious inhibitory effect on mycelial growth. After being treated with TDDE, offshoots of the mycelia increased, hyphal tips were contorted, and the cytochylema extravasated (Figure 6(A2-1–A2-2)). The SEM images showed that hypha in the TDDE treatment group significantly shrank, indicating significantly decreased activity of *B. cinerea* (Figure 6(B2-1–B2-3)). To determine internal changes in hypha, cross and vertical sections of *B. cinerea* hypha were observed by TEM. In the blank control, the internal organelles of mycelia were neatly distributed, the ridges of mitochondria were clear, and the nucleus was evenly distributed (Figure 6(C1-1–C1-5)). However, in the TDDE treatment group, the internal structure of cells was disordered, the ridges of mitochondria were fuzzy, and internal vacuolation was observed (Figure 6(C2-1–C2-5)), indicating that TDDE treatment had a significant effect on the internal organelles of *B. cinerea*. The drastic external and internal changes demonstrated that the activity and infectivity of *B. cinerea* was significantly decreased, which also explains why TDDE affected the toxicity of *B. cinerea* in vitro and in vivo.

### 2.5. Effect of TDDE on the Physiological Activities of B. cinerea 

Cell membrane permeability: The effects of different concentrations of TDDE on membrane permeability of *B cinerea* mycelia was observed by measuring the relative electrical conductivity (REC) of mycelia. The results showed that the REC of mycelia of *B. cinerea* in the blank control group and the TDDE treatment groups increased gradually over time (Figure S2A). The RECs of mycelia in the treatment groups were higher than that of the blank control group after 35 min, indicating that there was more electrolyte extravasation in mycelia cells after TDDE treatment, resulting in increased membrane permeability.

pH value and oxalic acid content: The pH and oxalic acid content of mycelia were measured using a PDB liquid-shaking flask method. The results showed that the pH values of the control and treatment groups decreased significantly and then increased over time. Meanwhile, the pH values of the treatment groups decreased more dramatically after four days’ culture and remained much lower than the pH value of the control group (Figure S2B). The oxalic acid content in the control and treatment groups increased at first then decreased, but the oxalic acid content in the treatment groups was significantly higher than that in the control group after four days’ culture (Figure S2C), which may contribute to the observed differences in pH values. The increased oxalic acid contents and decreased pH values in the treatment groups may contribute to the toxicity against *B. cinerea*, but the actual toxicity determined in vivo and in vitro was significantly decreased, indicating the excellent antifungal activity of TDDE.

Malondialdehyde (MDA) content: The level of MDA is used to determine the level of lipid oxidation. Compared with the control group, MDA content in the treatment groups increased at first and then decreased with increasing TDDE concentration, but all were significantly decreased relative to the control (Figure S2D). The results indicated that TDDE had a great effect on the membrane structure of *B. cinerea*, which resulted in the decrease of lipid oxidation levels in the membrane.

Antioxidant enzyme activities: SOD, POD, and CAT are superoxide anion scavenger enzymes, which play important roles in the biological antioxidant system. In the present study, SOD and CAT (except treatment EC_50_) contents increased significantly after TDDE treatments, while POD content changed minimally (Figure S2E–G). It is speculated that TDDE produced toxic effects against *B. cinerea* mycelia, stimulating the increased production of its defense enzymes.

Glycerol content: Total glycerol content is a strong osmotic regulatory substance of mycelia in pathogenic bacteria. After shaker fermentation of mycelia with EC_20_ = 0.475 μg/mL TDDE for 72 h, the accumulation of glycerol in mycelia reached 4141.56 μg/g, which was significantly higher than that of the blank control; the glycerol content decreased with the increasing concentration of TDDE (Figure S2H).

### 2.6. Antibacterial Activity of TDDE

**Figure 7:**
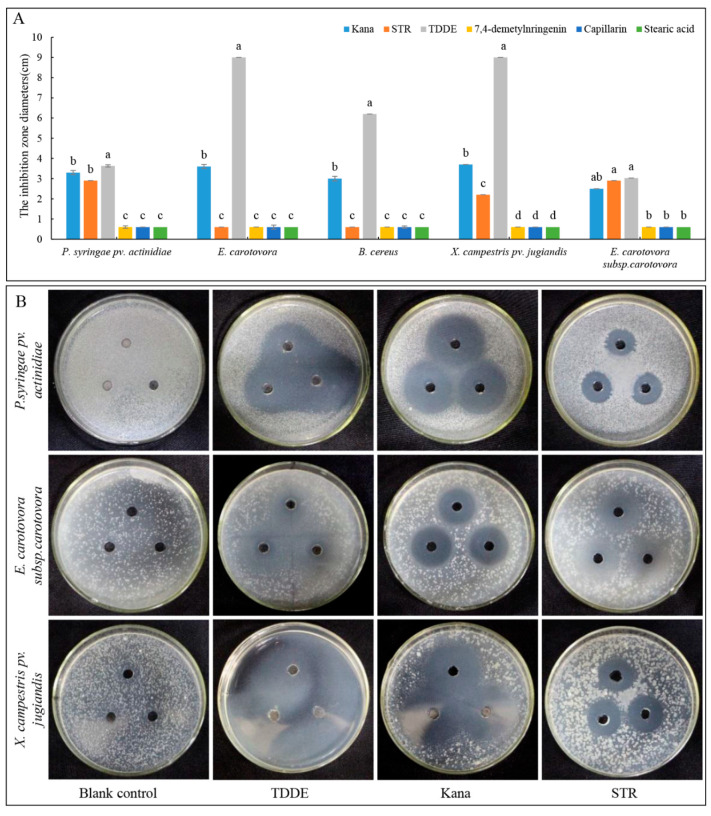
Antibacterial activities of compounds isolated from *A. ordosica*. (**A**) Antibacterial activities of four compounds at a concentration of 1.0 mg/mL; and (**B**) inhibitory effects of TDDE on different bacteria in culture. Kana, kanamycin; STR, streptomycin. The concentration used for all treatments in this assay was 1.0 mg/mL. Different lowercase letters in the figures indicate significant differences at *p* < 0.05 among different compounds against the same strain.

Antibacterial bioactivity assay in dish: Five bacterial species were used to test the activities of the isolated compounds, and the results showed that the compound TDDE had strong inhibitory effects on all tested bacteria, while the other compounds exhibited no antibacterial activity (Figure 7A). The antibacterial activity of TDDE was significantly higher than kanamycin (Kana) and streptomycin (STR), with the minimum inhibitory concentrations (MICs) against *P. syringae* pv. *actinidiae* (MIC = 34.593 μg/mL), *E. carotovora* (MIC = 59.184 μg/mL), *B. cereus* (MIC = 68.495 μg/mL), *X. campestris* pv. *jugiandis* (MIC = 73.074 μg/mL), and *E. carotovora* subsp. *carotovora* (MIC = 62.165 μg/mL) (Figure 7A,B; Table S1).

**Figure 8:**
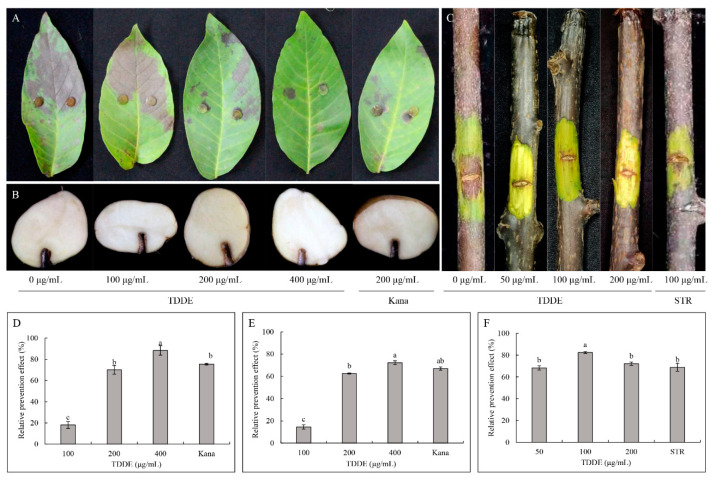
Effect of TDDE against different pathogenic bacteria on plant tissues. (**A**,**D**) Relative prevention effects against *X. campestris* pv. *jugiandis* on walnut leaves; (**B**,**E**) relative prevention effects against *E. carotovora* subsp. *carotovora* on potato; and (**C**,**F**) relative prevention effects against *P. syringae* pv. *actinidiae* on kiwifruit branches. Different lowercase letters in the figures indicate significant differences at *p* < 0.05.

Effect of TDDE on detached plant tissues infected with different pathogenic bacteria: The results of TDDE toxicity assays against different bacterial species revealed it had an 88.31% relative prevention effect against *X. campestris* pv. *jugiandis* on walnut leaves at a concentration of 400 μg/mL (Figure 8A,D), 72.18% relative prevention effect against *E. carotovora* subsp. *carotovora* on potato at a concentration of 400 μg/mL (Figure 8B,E), and 82.50% relative prevention effect against *P. syringae* pv. *actinidiae* on kiwifruit branches at a concentration of 100 μg/mL (Figure 8C,F). Meanwhile, this assay also demonstrated that the antibacterial activities of TDDE were significantly higher or equal to the positive controls (Kana or STR) for several bacterial species and plant tissues, which showed its excellent efficacy and broad-spectrum antibacterial activities (Figure 8).

## 3. Discussion

In this study, bioassay-guided isolation from the traditional Chinese herbal medicinal crop, *A. ordosica* (Artemisia, Asteraceae), was employed and proved to be an excellent method to isolate new bioactive phytochemicals. Although these four compounds were not new, the antifungal and antibacterial bioactivities of TDDE are described here for the first time. With respect to its uses in traditional Chinese herbal medicine for thousands of years [20,24,29], TDDE and the crude extracts of *A. ordosica* should be safe to humans and the environment. Therefore, they have great potential to be developed as novel low cost, high efficacy, and environmentally friendly botanical fungicides. Even so, the toxicity of TDDE towards humans and animals needs to be further verified before practical application. TDDE is a kind of polyacetylenes. Polyacetylenes are widely distributed in the genus Bupleurum of the Apiaceae family and has high toxicity [30]. Five polyacetylenes were isolated from *A. ordosica* in 2017, and they had repellent and fumigant activities against *Tribolium castaneum* [25]. Therefore, TDDE has great potential to be developed as a novel fumigant and fungicide, but the safe quantity, concentration, and application time need to be further investigated.

The 13 fungi used in this study are major pathogenic microorganisms on agricultural and horticultural crops, and all were significantly inhibited by *A. ordosica* crude extract and TDDE. For instance, *B. cinerea* is a worldwide occurring plant pathogen, causing pre- and postharvest gray mold rot on a large number of fruit, vegetables, and flower crops [31,32,33,34]. *T. cucumeris* is among the most important soil-borne pathogens all over the world, causing leaf, root, and fruit blight and rot on rice, tomatos, beans, okra crops, and more [35,36,37]. In the present study, virulence curves showed that TDDE displayed strong fungicidal activity against *B. cinerea* and *T. cucumeris* with EC_50_ values of 1.401 μg/mL and 0.464 μg/mL, respectively (Figure 4B and Table 2), while the EC_50_ values of MBC against *B. cinerea* is 7.40 ± 1.68 [38]. Due to the drug-resistance development, MBC has thus rarely been used in the past decade in China [39]. Furthermore, in this study the antifungal bioactivity assay in vivo demonstrated that the relative preventative effects of TDDE on tomato and strawberry plants against *B. cinerea* reached 84.11% and 96.37%, respectively (Figure 5D,E,G). These results indicated that TDDE has significant potential to be developed as a broad-spectrum fungicide.

TDDE is an excellent broad-spectrum fungicide against not only pathogenic fungi, but also a variety of pathogenic bacteria. Eleven pathogenic bacteria were used in this study, five of which were significantly inhibited in in vitro and in vivo assays (Figure 7), including *X. campestris* pv. *jugiandis* (cause of black rot in walnut) [40]), *E. carotovora* subsp. *carotovora* (cause of soft rot in potatoes and vegetables) [41,42]), and *P. syringae* pv. *actinidiae* (cause of bacterial canker in kiwifruit) [43]). All the relative prevention effects were higher than 70%, which was equal to or significantly higher than the positive controls Kana or STR (Figure 8). These data indicated that TDDE is an excellent broad-spectrum fungicide, and has extensive applications in disease control of agricultural and horticultural crops.

Knowledge of TDDE’s functions are very limited. It has a cis isomer, CDDE (cis-dehydromatricaria ester), which was found in *A. ordosica* previously [25]. Both of them are polyacetylenes, whose structures contain two or more triple bonds. Polyacetylenes are a group of secondary metabolites enriched in Araliaceae and Asteraceae plant families [25,44]. They have antifungal, antibacterial, serotogenic, allergenic, neurotoxic, cytotoxic, and anti-inflammatory properties [45,46,47]. Bioactive polyacetylenes (=polyynes) produced by these plants typically consist of aliphatic chains with several C–C triple bonds and serve plants in pathogen defense. In this study, it was firstly found that TDDE had excellent antifungal activities in vitro and in vivo, but the related mechanism and the function of chemical groups or bonds need to be further explored. In other studies, CDDE exhibited the strongest insecticidal activity (LC_50_ = 4.06 mg/L), and the oxygenous groups connected to the acetylenic bonds might contribute to its toxicity [25]. Therefore, TDDE probably has insecticidal activity and anticancer properties, which remains to be further investigated.

## 4. Materials and Methods

### 4.1. Plant Materials and Pathogens

#### 4.1.1. Plant Materials

The aerial parts of *A. ordosica* were collected from Yulin city in Shaanxi province, China, and identified by Prof. Li Y (College of Life Science, Northwest A&F University). A voucher specimen (NO.TGJ2017001) was preserved in Research & Development Center of Biorational Pesticide, Northwest A&F University. The dried herbs were smashed into powder before extraction.

#### 4.1.2. Pathogens

The tested strains were collected and identified from different areas of China and were preserved in the laboratory of the Research & Development Center of Biorational Pesticide, Northwest A&F University. These strains were also identified and used in the earlier studies [7,33,48], and the specific information is as follows:

Pathogenic fungi: *Alternaria alternate*; *Alternaria solani*; *Botryosphaeria dothidea*; *Botrytis cinerea*; *Colletotrichum gloeosporiodes*; *Cytospora* sp.; *Exserohilum turcicum*; *Fusarium graminearum*; *Fusarium oxysporum*; *Gaeumannomyces graminis*; *Magnaporthe oryzae*; *Phycomycetes*; *Phytophthora infestans*; *Rhizoctonia cerealis*; *Sclerotinia sclerotiorum*; *Thanatephorus cucumeris*.

Pathogenic bacteria: *Bacillus cereus*; *Bacillus subtilis*; *Erwinia carotovora*; *Erwinia carotovora* subsp. *carotovora*; *Escherichai coli*; *Pseudomonadaceae*; *Pseudomonas syringae* pv. *actinidiae*; *Ralstonia solanacearum*; *Salmonella*; *Staphylococcus aureus*; *Xanthomonas campestris* pv. *jugiandis*.

### 4.2. Extraction and Isolation of Bioactive Compounds

The ground-dried aerial parts of *A. ordosica*. (10 kg) were extracted with 80% ethanol (EtOH) (3 × 20 L, 24 h each time) at room temperature (20–25 °C) until exhaustion. The yellow viscous residue (1.2 kg) was filtered and concentrated under reduced pressure, dispersed in water, and sequentially extracted by petroleum ether (PE), chloroform (CHCl_3_), Ethyl acetate (EtOAc), and N-butyl alcohol (N-BuOH). The PE-soluble fraction (100 g) was subjected to silica gel column chromatography (120 × 1400 mm, Qingdao Marine Chemical Plant, China) eluting first with PE-Acetone (stepwise 200:1, 100:1, 90:1, 80:1, 40:1, 20:1, 15:1, 10:1, 5:1, 2:1, 1:1, 0:1, each 3 L) and finally methyl alcohol (MeOH) (3 L). Eleven corresponding fractions were obtained of H1 (3.45 g), H2 (4.68 g), H3 (7.51 g), H4 (11.03 g), H5 (6.13 g), H6 (21.00 g), H7 (17.00 g), H8 (6.20 g), H9 (17.00 g), H10 (3.25 g), and H11 (8.61 g) by thin layer chromatography analysis (TLC, Shanghai Spectral Instrument Co., Ltd., Shanghai, China). EtOH, PE, CHCl_3_, EtOAc, N-BuOH, and MeOH analytical reagents were purchased from the Adamas Company, Shanghai, China.

H3 was chromatographed on a silica gel column eluting with PE-Acetone (stepwise, 200:1, 100:1, 50:1, 20:1, 10:1, 5:1, 2:1, 1:1, 0:1, each 500 mL) to afford subfractions through a Sephadex LH-20 column using MeOH/CHCl_3_ (*V*/*V* = 1:1) and recrystallized to give compounds **3** and **4**. Compounds **1** and **2** were purified through a Sephadex LH-20 column (Walkman Company, Beijing, China) using MeOH/CHCl_3_ (*V*/*V* = 1:1) as eluent. These compounds were identified using Brucker DRX-500 MHz NMR (AVANCEIII, Brock, Switzerland).

### 4.3. Antifungal Activity Assays

#### 4.3.1. Antifungal Activity In Vitro and In Vivo

Effect of active fraction compounds on the mycelial growth of different fungal pathogens: The growth rate method was used to test the fungicidal activity of active fractions or compounds in dish [49]. Under aseptic conditions, 10 µL of the fraction or compound were added into 10 mL of PDA medium in a 50 mL centrifuge tube to make a 1/1000 concentrated solution, mixed well, and poured into a sterile 90 mm Petri dish, using distilled water as the blank control. A mycelial plug (6 mm diameter) obtained from the edge of 3–5 day-old colonies of each test fungus was placed in the center of the solidified medium, following methods described by [5]. Cultures were kept in a 25–28 °C incubator. The mycelia diameter was measured with reference to growth of the control, and the inhibition of mycelia growth was calculated using the formula [50]:

growth inhibition rate (%) = (colony diameter of control − colony diameter of the treatment)/(control colony diameter − 6) × 100% (1)

colony diameter (mm) = total colony diameter − 6 (dish diameter)(2)

Effect of active compound on the spore germination of *B. cinerea*: Treated growth media was prepared as described in the previous section. Plugs were taken from the edge of PDA medium with 0, 0.9, 1.8, 3.6, and 7.2 μg/mL of active ingredient or compound, and put on a wet filter paper. 25 μL of spore suspension (1 × 106 spores/mL) was dropped on each plug, cultured at 25 °C, and the spore germination rate during different growth stages was calculated using the following formula [51]:

spore germination rate (%) = (germination number/spore number) × 100%(3)

spore germination inhibition rate (%) = (spore germination rate of control − spore germination rate of the treatment)/spore germination rate of control × 100%(4)

Effect of active compound on detached plant tissues infected with *B. cinerea*: Tomato leaves and fruits, and strawberry leaves were used to test the activity of the active compounds in salver [51]. Collected plant tissue was washed with flowing water, after which the sample surface was swabbed using 75% EtOH. The different concentrations of the active compound (0, 12.5, 25, 50, 100, and 200 µg/mL, each with 0.5% of Tween-80) were directly sprayed onto the leaf surfaces. The MBC (100 µg/mL, methyl 2-benzimidazole carbamate; Purity 98%, Adamas Company, Shanghai, China) was used as the control. The detached plant tissues were punctured with holes with a diameter of 6 mm by the syringe needle. The 6 mm dish was taken from the edge of the tested fungus and placed in the region and incubated at 25 °C in moist conditions for about 3 days. The relative lesion area was measured using Image J software (https://imagej.nih.gov/ij/, accessed on 16 September 2021). Relative prevention effect was calculated according to the formula:

relative prevention effect (%) = (the incidence area of control − the incidence area of treatment group)/the incidence area of control × 100%(5)

Effect of active compound on tomato and strawberry plants infected with *B. cinerea*: Tomatoes and strawberry seedlings were planted in a pot and plants at the four-leaf stage were used in experiments. The active compounds, control, tested fungus, and statistical analysis were conducted using the same methods used above for leaves.

#### 4.3.2. Effect of Active Compounds on *B. cinerea* Mycelial Morphology

Light microscope analysis: Mycelial plugs (6 mm diameter), obtained from the edges of 3-day-old colonies of *B. cinerea* were placed mycelia-side down on the center of each PDA plate containing appropriate EC_50_ concentrations of active compound. Plates without active compound were used as blank controls. After 3 days incubation at 25 °C, these plates were then incubated in a thermostatic chamber. Samples of mycelium were cut from the edge of the tested fungus and put on a glass slide. Morphological changes of mycelia were observed by light microscope.

Scanning electron microscopy (SEM) analysis: SEM analysis was carried out using the methods described earlier [48,51]. Mycelial plugs (6 mm diameter), obtained from the edges of 3-day-old colonies of *B. cinerea*, exposed to the EC_50_ concentration of the active compound were fixed in 2.5% aqueous glutaraldehyde in phosphate buffer saline (0.1 M, pH = 7.2) for 4 h at room temperature. They were washed six times with the same buffer for 10 min, then dehydrated using a graded EtOH series (70%, 80%, 90%, and three times at 100%) for 30 min at each series. The fixed plug was treated with carbon dioxide critical point drying and gold coating in a sputter coater system, then observed using a JSM-6360LV SEM (JEOL Ltd., Tokyo, Japan).

Transmission electron microscopy (TEM) analysis: Mycelial plugs (6 mm diameter), obtained from the edges of 3-day-old colonies of *B. cinerea*, exposed to the EC_50_ concentration of the active compound were fixed in 2.5% aqueous glutaraldehyde in phosphate buffer saline (0.1 M, pH = 7.2) for 4 h at 4 °C, then fixed in 1% aqueous osmium tetroxide for 2 h at room temperature. They were washed six times with the same buffer for 10 min, then dehydrated using a graded EtOH series (30%, 50%, 70%, 80%, 90%, and 100%) for 30 min at each series. Next, open embedded and ultra-thin sectioning was carried out [52]. The samples were observed using a JEM-1200EX TEM (JEOL Ltd., Tokyo, Japan).

#### 4.3.3. Effect of Active Compounds on the Physiological Activity of *B. cinerea*

Cell membrane permeability: The electrical conductivity of mycelium was determined according to the reported method [51]. Five mycelial plugs (6 mm diameter), obtained from the edges of 3-day-old colonies of *B. cinerea*, were put into a 250 mL triangular flask containing 100 mL of PDB medium. After 24 h of liquid culture at 25 °C at 170 rpm, TDDE was added to the final concentrations of 2.0, 4.0, and 8.0 µg/mL. The flask without TDDE was used as a control. After 48 h, the mycelium was filtered and collected, washed twice with distilled water, and filtrated in a vacuum for 15 min. 500 mg fresh weight mycelia were weighed and suspended in 20 mL distilled water for 0, 5, 15, 45, 75, and 135 min, respectively, and measured using a CON510 conductivity meter (Eutech Instruments Pte Ltd., Singapore). After 135 min, the mycelia were boiled for 5 min and measured after natural cooling. The relative conductivity was calculated according to the formula (%):

(6) $$\mathrm{Relative}\;\mathrm{conductivity}\;\left(\%\right)=\frac{\mathrm{conductivity}\;\mathrm{at}\;\mathrm{each}\;\mathrm{time}\;\mathrm{point}}{\mathrm{final}\;\mathrm{conductivity}}\times 100\%$$

Oxalic acid content and pH value determination: Oxalic acid content was determined according to the method of a previous study [7]. Distilled water was used as control. After culturing on PDA plates for 4 days, the sclerotinia was punched along the edge of the colony with a hole punch (0.6 cm). Mycelial plugs with a diameter of 6 mm were inserted into a 250 mL conical flask containing 100 mL PDB, with each flask receiving 10 culture plugs. After liquid culture at 170 rpm and 25 °C for 72 h, TDDE was added to a final concentration of 3 g/mL without adding any additional reagent. As control, the culture medium was collected at 2, 4, 6, and 8 d and centrifuged at 8000 rpm for 10 min, and the supernatant used for detection. pH values were detected, and the absorbance of the sample at 620 nm was measured. The oxalic acid content was calculated with respect to the standard curve.

Malondialdehyde (MDA) and glycerol determination: The glycerol and malondialdehyde (MDA) content was determined using the commercial kits G0912F (Suzhou Geruisi, Suzhou, China) and BC0020 (Beijing Solarbio Science, Beijing, China) according to the kit instructions.

Determination of antioxidant enzyme activity: Superoxide dismutase (SOD), catalase (CAT), and peroxidase (POD) activities were determined using the following commercial kits, respectively, BC0170, BC0090, and BC0200, Beijing Solarbio Science.

### 4.4. Antibacterial Activity Assays

#### 4.4.1. Antibacterial Activity Assay in Culture

Bacterial suspensions were adjusted with distilled saline to a concentration of 1.0 × 10^5^ CFU/mL and stored at 4 °C. Inocula were screened for contamination by culturing on a solid medium. Each treatment was repeated three times, and each test was performed three times. Examined compounds were added (1.0 and 20 mg/mL) to 100 µL of LB medium) with bacteria inoculum, reaching the desired concentration in a microtiter plate to measure the minimum inhibitory concentration (MIC). The mixtures in microplates were incubated for 24 h at 37 °C on a rotary shaker. The MIC was defined as the lowest concentration at which there is no visible growth (at the binocular microscope level). Using a microplate manager, the optical density was measured at 655 nm and compared to blank and positive controls. The positive controls were 1.0 mg/mL kanamycin (Kana, Sigma-Aldrich, St. Louis, MI, USA) and streptomycin (STR, Sigma-Aldrich, USA), while 0.1% MeOH was used as the negative control. The melted LB medium was cooled to 45–50 °C and a 1.5% volume of the experimental pathogenic bacteria was added, mixed, and poured into sterile 90 mm Petri dishes (15 mL medium per dish). A 6 mm diameter hole was punched in the middle of the medium, and 30 μL 1.0 mg/mL fractions or compounds were added in it to test the antibacterial activity.

#### 4.4.2. Effect of Active Compound on Detached Plant Tissues Infected with Different Pathogenic Bacteria

The inoculation methods used were modified according to Ali and Reddy [53] and equally sized potato and walnut leaves and kiwifruit branches were used in this assay. The active compounds (0, 100, 200, and 400 µg/mL, containing 0.5% of Tween-80) were directly sprayed on the walnut leaves and, after drying, the bacteria plugs (diameter 6 mm) of *X. campestris* pv. *jugiandis* were fixed and inoculated using a needle and syringe. The stem of kiwifruit branches were cut into 2.5 cm long × 1 mm wide rectangular pieces. The active compounds (0, 50, 100, and 200 µg/mL, containing 0.5% of Tween-80) were applied to the branch pieces. After the agent was absorbed, 20 µL of *E. carotovora* (OD_600_ = 0.20) culture was inoculated into kiwifruit branches. The walnut leaves and kiwifruit branches were cultured in a crisper for moisture at 28 °C; water was used as the blank control, STR and Kana as the drug controls. For the water control, organizations around the rectangular area of the kiwifruit branch became significantly brown; the relative lesion area was quantified by Image J software and the inhibition of mycelia growth (relative prevention effect) was calculated according to the method in Section 4.3.1.

The active compounds were dissolved and absorbed by inoculation holes (10 mm diameter) that were punched in potato tubers. After drying, 20 µL of *E. carotovora* (OD_600_ = 0.20) were added into the inoculation holes. These potato tubers were cultured in the crisper for moisture at 28 °C; distilled water was used as the blank control, STR and Kana as the drug controls. For the water control, organizations around the rectangle became significantly soft, each treated decayed tissue sample was weighed separately. The relative prevention effect was calculated according to the following formula:

relative prevention effect = (1 − (weight of decay in water control group − weight of decay in treatment group)/weight of decay in water control group) × 100%(7)

### 4.5. Statistical Analysis

All the assays in this study had more than three biological replicates. Each biological replicate contained at least 15 plants, and only equally sized tissue samples were collected for the experiments. All data were analyzed with the Duncan test using SAS 9.2 software (https://www.sas.com/en_us/home.html, accessed on 16 September 2021). Different lowercase letters in the tables or figures indicate significant differences at *p* < 0.05.

## 5. Conclusions

In this study, four compounds were isolated from the H3 fraction in PE extracts of *A. ordosica* plants using a bioassay-guided method, and one of them (TDDE) was, for the first time, found to have excellent antifungal activities against six plant pathogenic fungi and five plant pathogenic bacteria. The plant tissue assays demonstrated that the relative preventative effects of TDDE against *B. cinerea* on tomato leaves, tomato fruit, and strawberry leaves reached 76.78%, 86.2%, and 80.89%, respectively. In pot experiments, the relative preventative effects on tomato and strawberry plants reached 84.11% and 96.37%. Microscope observations and physiological data indicated that TDDE had significant inhibitory effects on *B. cinerea* mycelial growth, including increasing top offshoot, contorting hyphal tips, and extravasating cytochylema. Meanwhile, it also had excellent relative prevention effects on walnut leaves (against *X. campestris* pv. *jugiandis*), potato tuber (against *E. carotovora* subsp. *carotovora*), and kiwifruit branches (against *P. syringae* pv. *actinidiae*). In conclusion, TDDE has excellent broad-spectrum antifungal activities, and it has great potential value to be used in developing new botanical fungicides.

## Figures and Tables

**Table 1:** Inhibitory effects of PE extraction on plant pathogenic fungi.

Fungus Name	Toxicity Regression Equation	EC_50_ Confidence Interval (μg/mL)	R^2^	χ^2^
*A. alternate*	Y = 2.538X − 7.153	658.462 (509.233 − 030.727) ^f^	0.969	1.633
*B. dothidea*	Y = 1.371X − 2.559	73.443 (60.976 − 134.885) ^a^	0.966	0.820
*B.cinerea*	Y = 4.669X − 12.425	458.382 (408.158 − 547.635) ^e^	0.986	0.671
*T. cucumeris*	Y = 1.843X − 3.455	75.007 (46.654 − 98.101) ^a^	0.973	1.115
*S. sclerotiorum*	Y = 3.276X − 7.533	199.276 (180.175 − 220.361) ^cd^	0.939	8.002
*F. graminearum*	Y = 2.484X − 5.773	210.765 (185.416 − 240.740)	0.953	3.873
*R. cerealis*	Y = 7.028X − 15.922	184.338 (139.056 − 240.433) ^c^	0.946	19.642
*G. graminis*	Y = 4.541X − 9.172	104.678 (77.664 − 126.254) ^b^	0.906	21.455
*E.turcicum*	Y = 1.154X − 3.287	703.142 (444.042 − 2397.242) ^g^	0.947	0.911
*Cytospora* sp.	Y =7.123X − 16.824	230.157 (215.675 − 245.254) ^d^	0.990	1.714

Note: These data were analyzed with the Duncan test; different lowercase letters indicate the significance difference at *p* < 0.05 among different strains.

**Table 2:** Inhibitory effects of TDDE on plant pathogenic fungi.

Fungus Name	Toxicity Regression Equation	EC_50_ Confidence Interval (µg/mL)	R^2^	χ^2^
*Cytospora* sp.	Y = 1.734 − 0.409X	1.722(1.466 − 2.019) ^e^	0.952	10.25
*B. cinerea*	Y = 2.95 − 0.432X	1.401(1.259 − 1.544) ^c^	0.997	0.709
*T. cucumeris*	Y = 0.724 + 2.175X	0.464(0.391 − 0.537) ^a^	0.974	11.684
*B. dothidea*	Y = 3.33X − 2.427	5.33(4.281 − 6.585) ^d^	0.977	59.627
*R. cerealis*	Y = 0.144 + 1.729X	0.826(0.702 − 0.956) ^b^	0.978	8.193
*G. graminis*	Y = 3.710X − 3.969	11.741 (8.361 − 17.123) ^f^	0.96	66.287

Notes: The data were analyzed with the Duncan test, and different lowercase letters in the table indicate the significance difference at *p* < 0.05 among different strains.

## Data Availability

Data is contained within the article and Supplementary Materials.

## Supplementary Materials

The following are available online at https://www.mdpi.com/article/10.3390/metabo11090629/s1, Figure S1: The H-NMR and C-NMR of TDDE, Figure S2: Effects of TDDE on *B. cinerea* physiological activities, Table S1: The minimum inhibitory concentration of TDDE against different bacteria.
