# Supplementary material for: Bioassay-Guided Isolation of Broad-Spectrum Fungicidal Active Compound from Artemisia ordosica
Source: Metabolites. 2021 Sep 17;11(9):629. doi: 10.3390/metabo11090629 (PMC8468595; doi:10.3390/metabo11090629)
Supplement: Supplementary file 1 [file metabolites-11-00629-s001.zip › metabolites-1371906-supplementary.pdf]

Supplemented materials:

Table S1. The minimum inhibitory concentration of TDDE against different bacteria.

Strain name	Minimum Inhibitory Concentration (MIC, μg/mL)
P.syringae pv. actinidiae	34.593
E. carotovora	59.184
B. cereus	68.495
X. campestris pv. jugiandis	73.074
E. carotovora subsp.carotovora	62.165

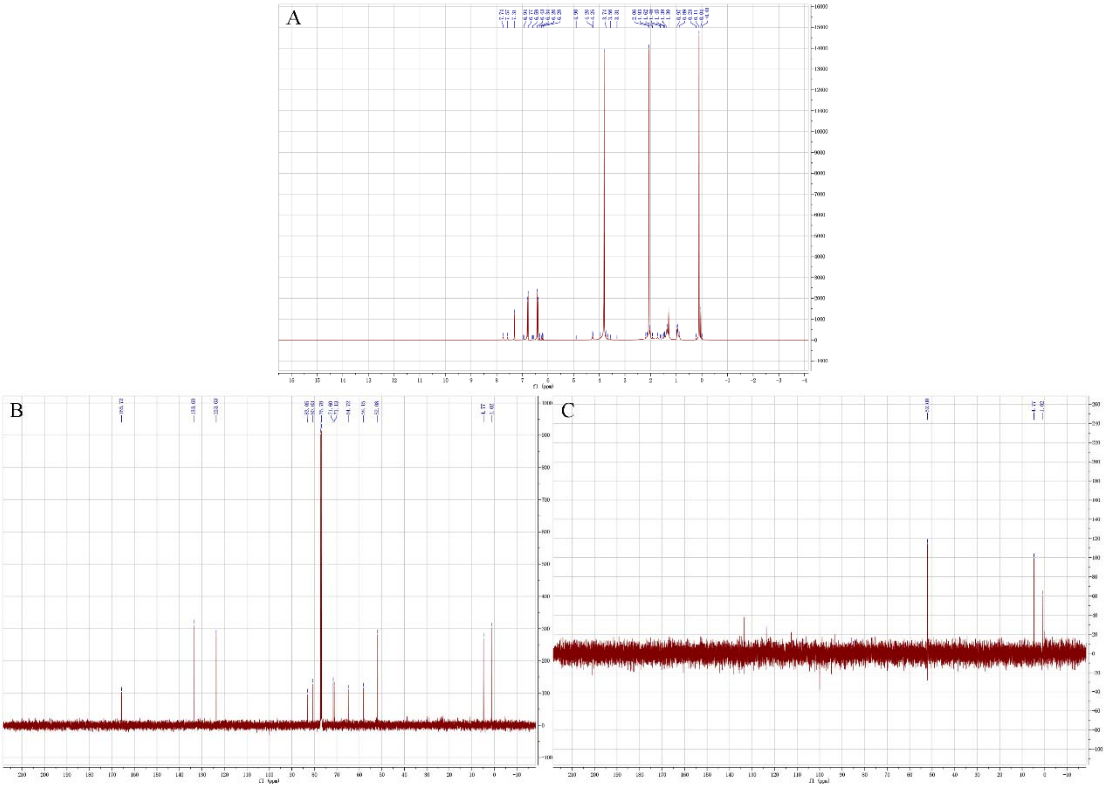

Figure S1. The H-NMR and C-NMR of TDDE. **A**. H-NMR; **B**. C-NMR; **C**. DEPT. NMR, nuclear magnetic resonance; DEPT, distortionless enhancement by polarization transfer.

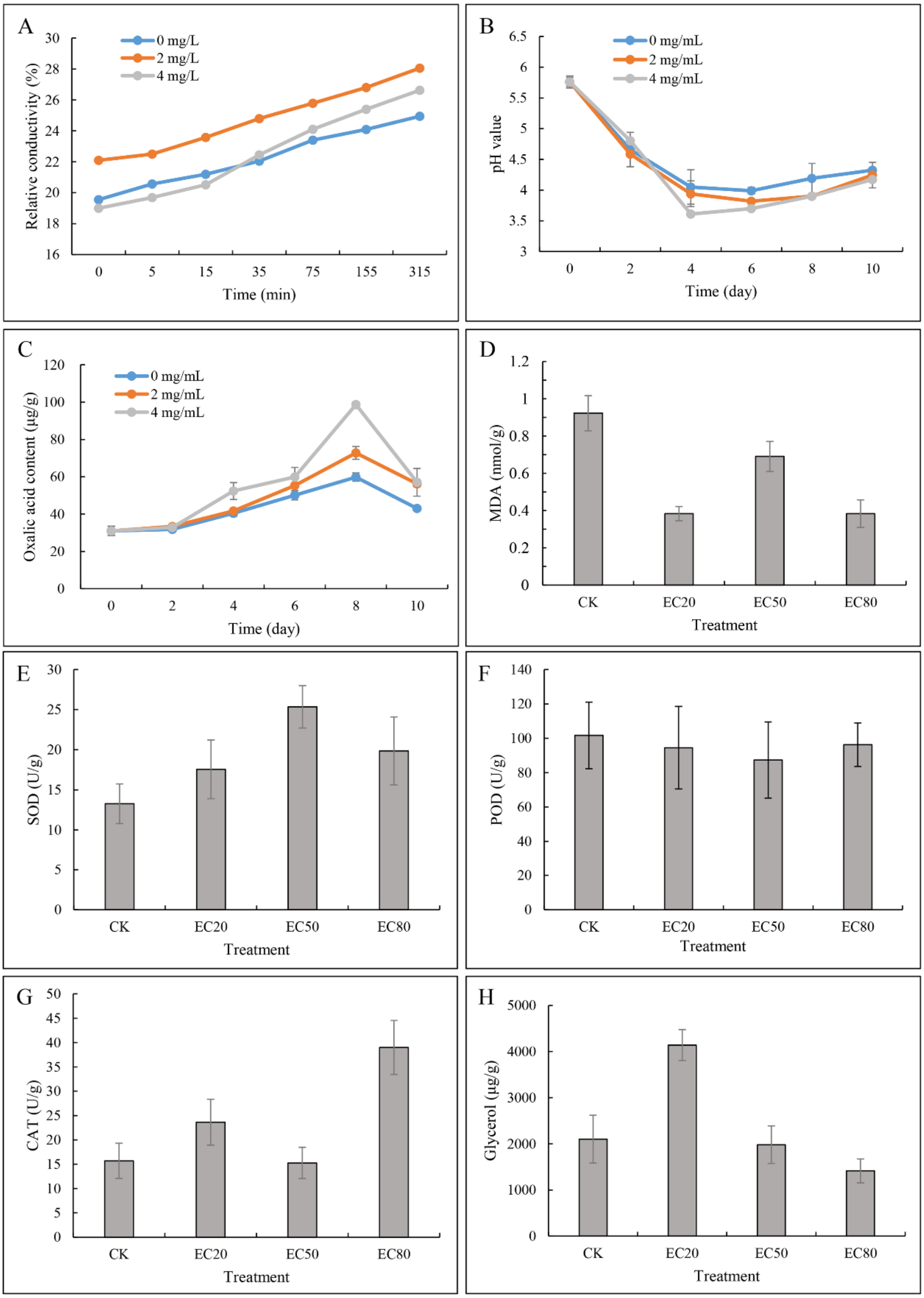

Figure S2. Effects of TDDE on *B. cinerea* physiological activities**. A**. Relative conductivity; **B**. pH value; **C**. Oxalic acid content; **D**. Malondialdehyde (MDA) content; **E**. Superoxide dismutase (SOD) activity; **F**. Peroxidase (POD) activity; **G**. Catalase (CAT) activity; **H**. Glycerol content. CK, blank control; EC20-80, different concentration of TDDE.
